# From leadership humility to customer satisfaction: a study of employee engagement in the Chinese service industry

**DOI:** 10.3389/fpsyg.2025.1564142

**Published:** 2025-11-03

**Authors:** Xinyang Zhang, Sanghyuk Yim

**Affiliations:** Dankook University, 152 Jukjeon-ro, Suji-gu, Yongin-si, Gyeonggi-do, Republic of Korea

**Keywords:** humble leadership, employee engagement, service performance, customer satisfaction, leadership humility

## Abstract

**Purpose:**

The study proposes to integrate Leader-Member Exchange (LMX) and Self-Determination Theory (SDT) to study Humble Leadership (HL) impact on Employee engagement, Service Performance and Customer satisfaction in the Chinese service industry.

**Design/methodology/approach:**

A quantitative approach used purposive sampling, including 485 participants from organizations in the Chinese service industry. The data was further processed and tested through a PLS-SEM procedure to check the hypothesized relationships.

**Findings:**

Results of the study prove that leadership humility (LHU; *β* = 0.224, *p* < 0.001), relational empowerment (REM; *β* = 0.186, *p* < 0.001), and psychological safety (PSA; *β* = 0.138, *p* = 0.003) could be identified as critical antecedents of employee engagement (EEN). Additionally, autonomy support (AUS; *β* = 0.135, *p* = 0.001), competence development (CDE; *β* = 0.345, *p* < 0.001), and relatedness fulfillment (REF; *β* = 0.126, *p* < 0.001) significantly influence engagement positively. Moreover, employee engagement (*β* = 0.757, *p* < 0.001) has a profound impact on service performance, which in turn dramatically affects customer satisfaction (*β* = 0.477, *p* < 0.001). The study confirms employee engagement (*β* = 0.406, *p* < 0.001) as a driver of service performance and customer satisfaction within the service industry context.

**Practical implications:**

The study results propose practical suggestions for managers and leaders in the service industry as Organizations should develop humble leadership behaviors, empowering, and creating psychological safety while satisfying employees’ needs for autonomy, competence, and relatedness.

**Originality/value:**

The study helps in defining humble leadership as it integrates LMX and SDT frameworks in the context of the Chinese service industry. The study thus provides empirical evidence as to how humble leadership and employee engagement jointly work in the direction of better service performance and customer outcomes, consequently contributing to the literature on both leadership and service management.

## 1 Introduction

The service industry has become one of the most important engines of economic growth in China (Nabar-Bhaduri and Vernengo, 2024) playing a significant role in employment and Gross Domestic Product (GDP) growth (Xin et al., 2023). As the sector evolves within an increasingly competitive and globalized market, the role of leadership in influencing organizational results is enormous (Afzal and Kalra, 2024). Service leadership is, therefore, not only responsible for driving the strategic agenda (Su, 2024) but also for creating a working environment that allows maximum employee engagement (Laureani et al., 2024). Employee engagement is defined as an approach to workers that accompanies their emotional commitment and involvement in the work process (Narayanasami et al., 2023), such engagement is time and again, positively associated with productivity, quality of service, and a decrease in turnover (Kossyva et al., 2023).

Humble leadership has emerged as a style in which the traits of humility, self-awareness, and empowering followers are paramount while offering a potential solution to contemporary disengagement problems in organizations (Kim et al., 2023). Prior research findings, largely based on samples drawn from Western contexts (Elhadidy and Gao, 2024; Zhang et al., 2024), indicate that humble leadership is positively related to team performance (Van Tongeren et al., 2023), psychological safety (Mrayyan, 2024), and employee engagement (Liu H. et al., 2024). Humble leaders are likely to enhance employee voice (Kim and Cho, 2023) and admit their limitations while seeking continuous learning (Chan et al., 2024), which could result in a more engaged and proactive workforce (Yao and Hao, 2023). In the context of the Chinese service industry, where high employee turnover and low engagement are persistent challenges (Zhu et al., 2023), understanding the leadership styles that can promote engagement is of both academic and practical significance (Liu and Nie, 2024).

Prior research has primarily focused on how humble leadership influences engagement specifically within the service sector (Zhang et al., 2024; Gao and Gao, 2024). Studies also take precedence over the other majority of studies that relate to transformational (Nordin et al., 2024; Asif et al., 2024), and ethical leadership styles (Arora and Arora, 2024) rather than humility as a leadership trait, which limits our understanding of the diverse leadership styles that can improve employee engagement (Jiatong et al., 2022). A limited number of studies have determined the linkage of humble leadership, showing the mediating effects through psychological safety, particularly in Chinese service industries (Liu et al., 2023b). While leadership styles interacting with autonomy support, competence development, and fulfillment of relatedness account for the combined effect in the service environment (Jung et al., 2023), this fact has often been overlooked in extant research (Burhan and Khan, 2023). However, a major gap remains in the understanding of how leadership-driven employee engagement affects customer satisfaction as an important outcome in the service sector (Zhao et al., 2023).

However, despite growing relevance, humble leadership remains underexplored in non-Western contexts (Bai et al., 2023; Tariq et al., 2023) such as the Chinese service industry, where leadership practices are often shaped by traditional hierarchical structures and cultural values such as guanxi (interpersonal relationships) (Kang et al., 2023) and collectivism (Tano et al., 2023). Thus, understanding how humble leadership influences employee engagement is of theoretical enrichment and practical significance (Khairy et al., 2023; Willett et al., 2023). This study endeavors to address this gap by investigating the correlation between employee engagement and humble leadership in the service sector of China. Specifically, the objectives of this study which is to examine how humble leadership influences employee engagement and, through intermediary mechanisms such as service performance, and customer satisfaction, enhances outcomes in the Chinese service industry, the following questions needs to be addressed:

1. How does humble leadership influence employee engagement in the Chinese service industry?
2. How do mediating factors, such as service performance and employee engagement impact customer satisfaction?

To answer the above questions, this study integrates Self-Determination Theory (SDT) (Deci et al., 2017) and Leader-Member Exchange (LMX) (Graen and Uhl-Bien, 1995), as few studies explored multifaceted influences on leadership in employee engagement and, subsequently, service performance (Han and Sears, 2023; Liu et al., 2023a). LMX theory focuses on the quality of the relationships between leaders and their subordinates. It postulates that high-quality exchanges result in higher levels of trust, respect, and mutual obligation, which could enhance employee engagement (Yang et al., 2023; Ribič and Marič, 2023). The basic psychological needs, autonomy, competence, and relatedness, associated with self-determination theory, look at increasing intrinsic motivation and engagement in employees (Ding and Kuvaas, 2023). This study combines the two concepts to determine how humble leadership affects employees’ engagement by considering the mediating role of LMX and satisfaction with basic psychological needs.

This study adds to the body of knowledge on leadership in the Chinese service industry and employee engagement. First, it fills in the gaps created by the relationship between humble leadership and employee engagement, as a concept widely overlooked against the other leadership styles of ethical and transformational leadership (Fareed et al., 2023). This study even further builds upon the scant literature on LMX theory and SDT integration in service industries, extending to more unique outcome analysis, a more integrative perspective, and more holistic insights into how leadership impacts employee engagement and performance (Liu J. et al., 2024). Third, this study provides empirical insights on how a humble style of leadership, in general, engenders psychological safety in employees, an important factor, but not deeply explored in the Chinese service sector context. The research also shows how humble leaders create psychological safety among employees through a psychological mechanism of protective surroundings (Abudureheman et al., 2023). Finally, it will address the critical link between employee engagement and customer satisfaction, providing practical implications for improving service performance in the highly competitive Chinese market (Leng and Tong, 2022).

## 2 Literature review and conceptual

Humble leadership has been characterized by self-awareness, openness to feedback, appreciation of others, and the acknowledgement of one’s limitation. This style of leadership underlines humility in terms of leaders being conscious of their weakness and looking for others’ input in trying to foster a learning culture and toward collaboration. Humble leadership has been established as influential in eliciting employee creative performance through boundary-spanning behavior but moderated by the traditionality of the workplace (Zheng and Ahmed, 2022). Similarly, team creativity is enhanced by leader humility, impacting team learning processes (Chen et al., 2021). Another study demonstrated that leader humility mediated work well-being, which in turn was mediated by psychological safety and error management climate (Zhang and Song, 2020). Ding et al. (2020) further explored humble leaders as they promote team innovation by fostering reflexivity and utilizing expertise diversity across the teams. Besides, humble leadership positively influences employee job performance by enhancing supervisor-subordinate relationships, more so when the perceived leader integrity is high (Yang et al., 2022). The effect of humble leadership on innovation has also been identified to be partially mediated by knowledge exchange and job complexity in the technology sectors (Jiang et al., 2019). On the whole, humble leadership is a key factor that affects innovation, creativity, and well-being in the respective Chinese service-based organizations.

Multiple previous researchers have studied humble leadership through multiple models, where Social Information Processing (SIP) Theory (Zettna et al., 2025); Mediated Moderation Model (Wang et al., 2024); Conservation of Resources (COR) Theory (Hoang et al., 2024); Moral Licensing Theory (Chen et al., 2024); Affective Events Theory (AET) (Feng et al., 2024) are studied. Additionally, servant leadership theory focuses on those who serve others and the cultivation of an enabling environment, which largely resonates with the principles of humble leadership (Bao et al., 2018). According to social exchange theory, effective mutual relations in a working environment can be developed between leaders and followers when humble leaders show a higher level of trust and commitment toward their followers for increased performance and innovative behaviors (Iqbal et al., 2021). Further, the self-determination theory emphasizes the needs of employees for autonomy, competence, and relatedness to be satisfied with the help of humble leadership that creates a supporting atmosphere at work (Kül and Sönmez, 2021).

Humble leadership shows a positive relationship with high employee engagement and innovative behaviors at work (Yang et al., 2019). Humble leadership has also been shown to elevate the position of employees within the organisation and help them develop their own leadership potential, lending further evidence that humility not only creates engagement, but also potential leaders within organisations (Lin et al., 2024). Organizational citizenship behaviors fostered by humble leaders are facilitated through mechanisms like the use of strengths and job crafting by their staff. Humble leadership in the context of creativity fosters team creativity via the facilitation of team learning and boundary-spanning behaviors; cultural factors such as traditionality further moderate these effects (Zheng and Ahmed, 2022). Further, humble leadership supports work well-being through the facilitation of a psychologically safe work environment that leads to increased performance of the employees (Zhang and Song, 2020). Lin and Tse (2025) pointed out that humble leaders not only behave wisely and touch the hearts of their followers-those they lead-but they purposefully encourage the development of their followers’ leadership ambition by empowering them to take initiative and become leaders in turn. In addition to this, humble leadership also results in the success of projects through increased team engagement; this is therefore applicable to organizational outcomes beyond just individual performance (Waseem et al., 2023). Other research also indicated that humble leadership enhances employees’ resilience and career success mechanisms through work-related promotion focus and innovative work behavior (Zhu et al., 2019; Chughtai and Arifeen, 2022). All these studies highlight the far-reaching benefits of humble leadership in enhancing employee outcomes across various sectors and organizational contexts in China.

Although the relationship between humble leadership and employee engagement has been relatively well established in the Western context, it is still being developed in the Chinese service industry. Few studies have examined how humble leadership interacts with cultural and industry-specific factors within China. Most existing studies also dwell more on the positive aspects of humble leadership, thereby leaving behind the negative effects in their studies. For instance, very humble leaders can rarely assert authority or be decisive, a characteristic that can easily lead to confusion and employees lacking direction (Owens and Hekman, 2016).

While research has been conducted independently on humble leadership in a few studies, the integration of LMX and SDT makes it a relatively new approach for explaining its impact on employee engagement. LMX theory offers a relational view of leadership that mainly deals with how leader behavior affects the quality of relationships with subordinates, while SDT is more of a motivational framework for understanding how leadership can fulfill employees’ psychological needs. It is within this integration of frameworks that the current study will further help us understand how humble leadership influences employee engagement in the Chinese service industry. The combination of LMX and SDT with humble leadership has received extensive empirical attention. Earlier studies have indicated that leadership styles oriented toward positive LMX tend to promote psychological need satisfaction and, hence, employee engagement. However, empirical analysis of this relationship is limited to the Chinese service industry.

**Figure 1:**
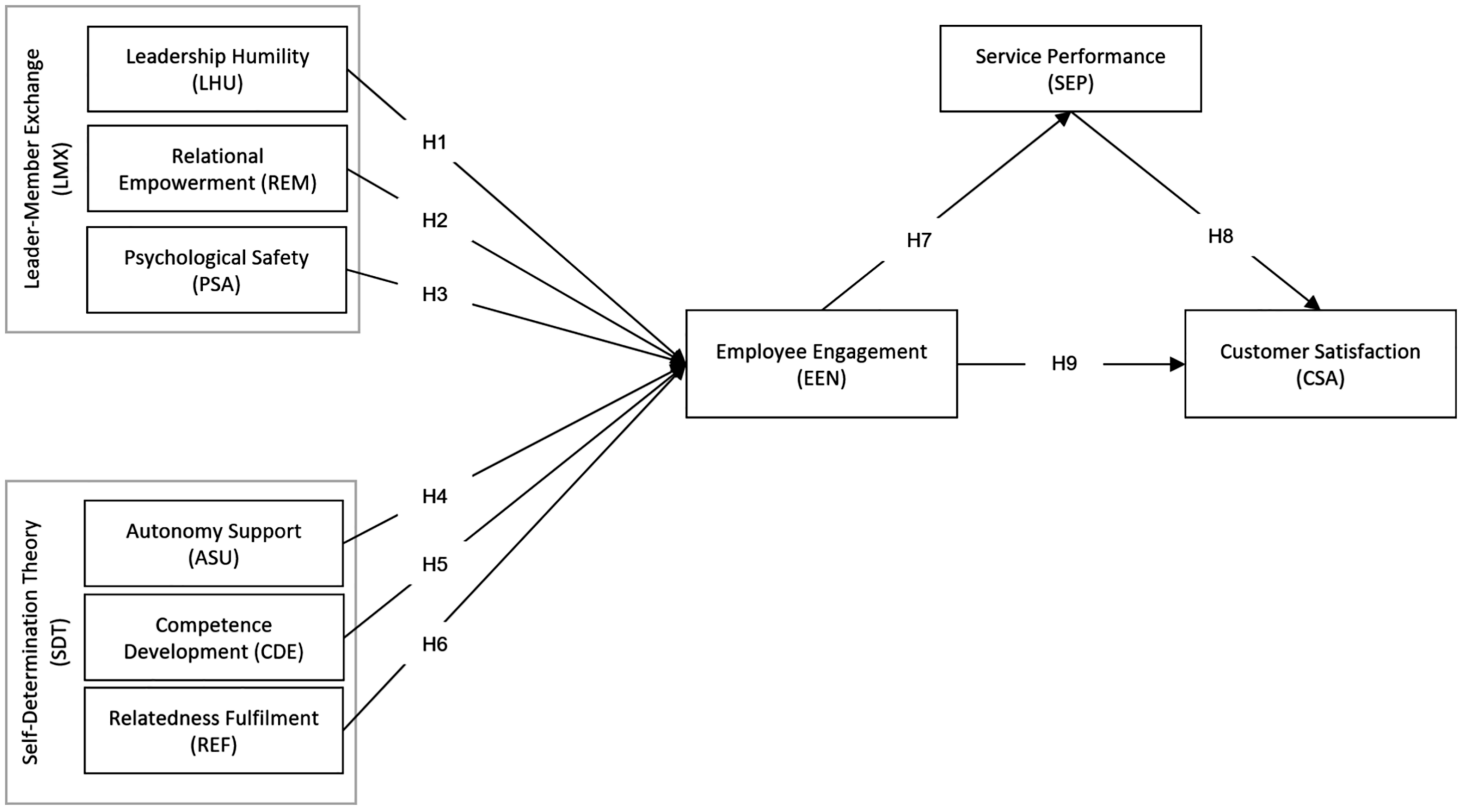
A proposed conceptual model (source: authors own creation).

**Figure 2:**
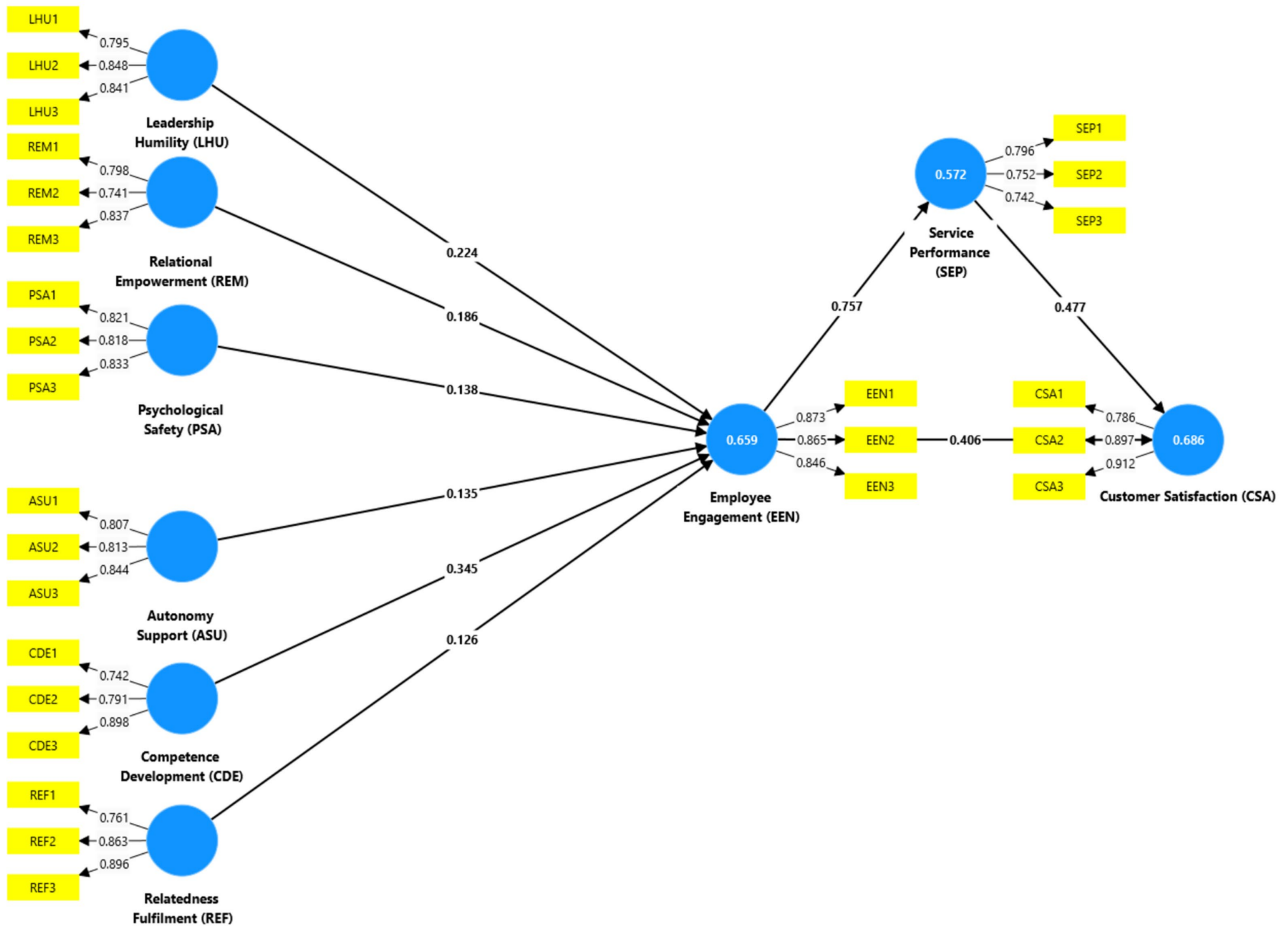
Structural model (source: authors own creationt, all of which contribute tn).

The study proposes to integrate the conceptual model in Figure 1 to study the humble leadership influence on employee engagement through LMX theory and SDT. Humble leaders are likely to create high-quality leader-member exchanges due to the influence of relational empowerment and psychological safety, which enhance employee psychological empowerment and perceived job meaningfulness, leading to work engagement. Specifically, humble leaders foster high-quality LMX by promoting relational empowerment, psychological safety, and humility in their interactions, instilling a sense of belonging. As per LMX theory, humble leadership enhances interpersonal relationships between leaders and employees, enabling smooth communication and collaboration, which further boosts engagement. Humble leaders may cultivate autonomy, competence development, and relatedness fulfillment by satisfying basic psychological needs and fostering higher levels of intrinsic motivation and engagement. Empirical evidence shows that when humble leaders provide autonomy support and guidance in competence development, employees become more engaged and contribute creatively toward organizational goals (Chen et al., 2021). Importantly, relational empowerment and psychological safety, which are central to humble leadership, create an environment where employees feel secure enough to share their ideas and take initiative, thus driving engagement and innovation (Zhang and Song, 2020) (Figure 2).

## 3 Hypotheses development

Leadership humility represents a leader’s willingness to acknowledge one’s limitations while appreciating the strengths and contributions of others, thereby fostering an open learning-oriented environment (Hanel et al., 2023). Recent empirical studies (Davis et al., 2023) have shown that humility in leadership creates a psychologically safe environment at work in which employees feel valued and respected, further stimulating their work engagement. In the context of the LMX theory, the quality of the relationship and interaction between leaders and employees greatly determines employees’ work attitudes and behaviors, including engagement (Tariq et al., 2023). Through increased trust, openness, and collaboration, a humble leader is likely to enhance high-quality leader-member exchanges, thereby increasing employee engagement (Yao and Hao, 2023). Leaders who exhibit humility are more likely to empower employees, provide them with autonomy, and facilitate competence development, all of which contribute to higher engagement. Therefore, it is hypothesized that

*H1*: Leadership Humility positively influences Employee Engagement

Relational empowerment is the process of enabling workers to be capable and motivated to take up tasks, make decisions, and have autonomy in their roles from a relational context (Panduwiyasa et al., 2024). It adds emphasis to the cooperative relation in which workers feel trustful and regarded and hence increases their engagement. These are brought about by participation in decision-making (Dennerlein and Kirkman, 2023). LMX theory suggests that efficient leader-member exchanges create feelings of being empowered among employees themselves, resulting in higher employee engagement behaviors (Gu et al., 2024). For instance, when a leader confers more job-related control and influence at work over his or her subordinates, such provision is consistent with the needs of employees’ autonomy and competence. So, it is hypothesized that

*H2*: Relational Empowerment positively influences Employee Engagement

Psychological safety involves shared perceptions of the work environment within a safe context of interpersonal risk-taking, such that employees feel free to talk about their ideas or questions and even voice their concerns without trepidation of being punished negatively (Pang et al., 2023). Based on the premise that high-quality relationships between leaders and employees create a positive context in the workplace, LMX theory suggests that, in turn, this prepares the ground for psychological safety and, ultimately, higher levels of employee engagement (Martin et al., 2023). Psychological safety also meets the intrinsic needs of employees for relatedness, autonomy, and competence, and hence is an important determinant of fostering intrinsic motivation and engagement (Khairy et al., 2023). Therefore, it is hypothesized that.

*H3*: Psychological Safety positively influences Employee Engagement.

Autonomy support can be defined as the extent to which leaders motivate self-initiation among employees and grant employees a choice in their jobs, thereby fostering a sense of volitional effort among employees (Shih et al., 2019). Autonomy support is a critical component of creating intrinsic motivation. It directly boosts employee engagement through satisfaction of employees’ psychological need for autonomy (Zhou et al., 2019). Further support for this relationship is shown by leader-member exchange, which argues that leaders who grant autonomy and support are bound to offer high-quality exchanges with their employees and hence higher engagements (Shih et al., 2019). Autonomy is one of the fundamental psychological needs that must be satisfied for individuals to experience intrinsic motivation and well-being, both of which are critical components of employee engagement. Thus, it is hypothesized that

*H4*: Autonomy Support positively influences Employee Engagement

Competence development involves development opportunities for employees to develop new skills, improve their capability, and attain career growth in the organizational context (Liang et al., 2020). According to the Self-Determination Theory, competence is one of the basic psychological needs whose satisfaction leads to increased levels of intrinsic motivation for action (Zhou et al., 2019). This would make them have a feeling that they are empowered and competent enough, encouraging intrinsic motivation to get involved in many activities since they are certain of their capacity for the psychology of empowerment. These employees are perceived as more powerful, with high competence and self-efficacy in the organisation. The Leader manager exchange theory suggests that by facilitating skill development and resourceful growth support, leaders build stronger relationships with employees and enhance their engagement (Shih et al., 2019). Therefore, it is hypothesized that

*H5*: Competence Development positively influences Employee Engagement

Relatedness fulfillment is the satisfaction of an inborn need to establish important connections and relationships with others in one’s work (Chen et al., 2023; Chen et al., 2023). Self-determination theory postulates relatedness as one of the three basic psychological needs that, when satisfied, enhance intrinsic motivation and engagement in a task (Wu et al., 2017). Engaged employees experience relatedness fulfillment, a sense of belonging, and connections with colleagues that arise by creating a supportive and collaborative work environment (Miao et al., 2014). Based on LMX theory, leaders develop very strong relational bonds with their employees, and they contribute to higher levels of relatedness, which in turn influences higher engagement (Liu et al., 2023a). Therefore, it is hypothesized that

*H6*: Relatedness Fulfillment positively influences Employee Engagement

Employee engagement is defined as a positive, fulfilling, work-related state of mind comprising vigour, dedication, and absorption in work activities (Ali et al., 2022). Engaged employees are more enthusiastic and committed to the roles they play and are highly energetic in providing their services, which directly results in high service performance (Liu et al., 2022). This is underpinned by previous studies (xxx) which argue that whenever workers feel engaged and supported by their organisation, they normally respond with increased levels of performance (Chen and Peng, 2019). Empirical studies by Lambert et al. (2021) have been able to establish that engaged employees are more productive and deliver the best customer service which leads to enhanced performance outcomes. Therefore, it is hypothesized

*H7*: Employee Engagement positively influences Service Performance

Service performance is the manner in which employees deliver their services effectively of high quality, and to the satisfaction of the desires, needs, and expectations of a customer; that is, either meeting them or going beyond them (Yang et al., 2020). In light of previous studies (Su et al., 2019), right from the point where service performance exceeds one’s expectations, they get satisfied and thus their reactions to the performance become positive, leading to loyalty. There is evidence in many studies that there is a strong positive relationship between service performance to satisfaction (Qing et al., 2023). Studies also established that high-performance employees with excellent service delivery had a strong positive relationship that affects the degree of customer satisfaction (Ling et al., 2016). The SERVQUAL model, on the other hand, identifies the three most critical factors that affect customer satisfaction: reliability, responsiveness, and empathy in service delivery (Adhikari et al., 2023). Therefore, it can be hypothesized that

*H8*: Service Performance positively influences Customer Satisfaction

Employee engagement is a positive state of work well-being, characterized by vigour, dedication, and absorption in their job (Andrlić et al., 2023). More motivated, higher-performing, and quality-oriented employees are those who are more engaged, as seen in previous studies (Xu et al., 2023); these factors add up to higher levels of customer satisfaction. Engaged employees are most likely to engage in proactive behaviors, perform beyond formal job expectations, and create favorable interactions with customers, thereby improving service quality delivery, which results in customer satisfaction (Jiang et al., 2023). This is further suggested that higher internal service quality through engaged employees delivers higher external service quality, and hence, yields better customer satisfaction (Wang et al., 2023). Therefore, it is hypothesized that,

*H9*: Employee Engagement (EEN) positively influences Customer Satisfaction (CSA).

## 4 Research methodology

This research employed a positivist research philosophy and study design which was mainly a deductive reasoning logic as the core purpose was to determine hypothesized relationships between leadership behaviors, psychological need satisfaction, employee engagement, service performance and customer satisfaction. This has added objectivity to the study which is essential in finding quantifiable evidence through structured measurement and statistical analysis (Saunders et al., 2019).

The study employed a purposive sampling similar to previous studies (Gupta et al., 2024; Raziq et al., 2024) in the selection of respondents with relevant experience in the service industry. Whilst probability sampling may have improved generalisability, purposive sampling was deliberately adopted to record the views of employees directly engaged in frontline service delivery, since they are the most appropriate population against which to examine leadership, engagement and customer outcomes. Ensuring the sample became appropriate to fulfill the study objectives. A sample of 485 respondents was selected from different service-oriented organisations around key regions, such as Beijing, Shanghai, and Guangdong. This was selected as these three regions are regarded as the most important in terms of economics and the centres where activities in the service industries are concentrated (Ren et al., 2022). Contextually, 650 questionnaires were distributed, of which 517 were returned. After screening for completeness and data quality, 485 usable responses were retained, resulting in a usable response rate of 74.6%. Based on Cohen’s (2013) suggestion on the use of SEM, a model with several latent variables should have a minimum sample size of 200. The justification of the sample size was further checked using G*Power analysis, which assumes a required power level of 0.95 and a significance of 0.05, to detect medium effect sizes (f^2^ = 0.15). In addition, the minimum required number of respondents was 350; hence, having 485 respondents was a clear solution to the final sample of this robust statistical analysis.

Further, the pilot study was done using a questionnaire that was tested with 50 employees in the service industry to measure clarity, reliability, and validity. From the test results, ambiguity or any other discrepancies in the questionnaire items could be pointed out by the research team. Through feedback from the pilot, the minor revisions made gained clarity and appropriateness in the questions. Reliability was examined using Cronbach’s alpha to test the reliability of the scales used in the pilot study mentioned in Table 1. All constructs had threshold values higher than the 0.70 threshold.

**Table 1:** Discriminant validity (source: authors own creation).

Construct	Cronbach’s alpha	Composite reliability	Average variance extracted
ASU	0.764	0.789	0.675
CDE	0.760	0.897	0.661
CSA	0.833	0.848	0.752
EEN	0.826	0.832	0.742
LHU	0.771	0.773	0.686
PSA	0.766	0.774	0.679
REF	0.793	0.789	0.709
REM	0.706	0.718	0.629
SEP	0.757	0.753	0.583

Data was collected using a structured questionnaire designed to measure constructs of interest. The questionnaire was based on scales established from the previous literature mentioned in Table 2 and consisted of sections focusing on leadership behaviors, psychological need satisfaction, employee engagement, service performance, and customer satisfaction. Autonomy Support (ASU) was measured by using items jointly adopted from Liu et al. (2020) and Shih and Chen (2015). Competence Development (CDE) was measured using scales by Adie et al. (2008). Customer satisfaction was measured in accordance to Shih and Chen (2015), in which customer satisfaction is operationalized in terms of service performance. Employee Engagement Order Domains rated according to Embalsado et al. (2025) were selected as employee engagement (EEN) items. Leadership Humility (LHU) Scale was measured by Owens and Heckman (2012). Edmondson (1999)'s key measure of Psychological Safety (PSA) was used. Relatedness Fulfillment (REF) was indicated by items from the questionnaire of Frielink et al. (2024). Relational Empowerment (REM) was based on the widely used scale by Spreitzer (1995). Finally, a Service Performance (SEP) construct was measured using instruments that had been adapted from Shih and Chen (2015). Contextually, no control variables were included in the analysis, as the focus was on the direct relationships among the core constructs. The statements were evaluated by the respondents using a seven-point Likert scale, with responses ranging from “strongly disagree” to “strongly agree.” Furthermore, demographic data was gathered to establish a context for the sample referenced in Table 3.

**Table 2:** Hypothesis results (source: authors own creation).

Hypotheses	Path	Standard deviation	T statistics	*p* values	Path coefficients	Results
H1	LHU → EEN	0.050	4.502	0.000	0.224	Supported
H2	REM → EEN	0.034	5.387	0.000	0.186	Supported
H3	PSA → EEN	0.047	2.941	0.003	0.138	Supported
H4	ASU → EEN	0.041	3.333	0.001	0.135	Supported
H5	CDE → EEN	0.037	9.228	0.000	0.345	Supported
H6	REF → EEN	0.032	4.010	0.000	0.126	Supported
H7	EEN → SEP	0.016	6.690	0.000	0.757	Supported
H8	EEN → CSA	0.040	10.047	0.000	0.406	Supported
H9	SEP → CSA	0.043	11.168	0.000	0.477	Supported

**Table 3:** Customer demographic (source: authors own creation).

Variable	Category	Frequency (*n*)	Percentage (%)
Gender	Male	228	47.00
	Female	257	53.00
Age	18–24 years	94	19.40
	25–34 years	150	30.90
	35–44 years	132	27.20
	45–54 years	66	13.60
	55 + years	43	8.90
Education Level	High School or below	65	13.40
	Bachelor’s Degree	232	47.80
	Master’s Degree or higher	188	38.80
Occupation	Student	72	14.80
	Employed (Full-time)	268	55.30
	Employed (Part-time)	59	12.20
	Self-employed	30	6.20
	Unemployed/Other	56	11.50

Surveys were administered through online and offline modes to cover as much area as possible within selected regions. Online surveys were conducted through email and professional networking websites, and offline surveys were conducted in workplaces with the support of the Human Resources Department. This approach guarantees broad-based participation from employees working in diverse service sectors such as hospitality, retail, financial services, and customer support.

## 5 Result

### 5.1 Demographic profiles

Below are the details for the respondents in Table 3.

### 5.2 Reliability and validity

These constructs were verified by the measurement model, which was rigorously evaluated for reliability and validity. Cronbach’s alpha and Composite Reliability (CR) were implemented to guarantee reliability testing. All constructs exhibited satisfactory reliability, as Cronbach’s alpha values for each construct exceeded the predetermined threshold of 0.70, suggesting a suitable level of internal consistency. The reliability of the measurement instruments is demonstrated by the fact that the CR value for all dimensions exceeds the cut-off mark of 0.70, as shown in Table 1. The Average Variance Extracted (AVE) was used to establish convergent validity. The constructs’ AVEs were all greater than 0.50, suggesting that latent constructs accounted for more than 50% of the variance in the indicators.

The Fornell-Larcker criterion and the heterotrait-monotrait (HTMT) ratio were employed to evaluate discriminant validity. The Fornell-Larcker criterion was satisfied, as the square root of AVE for each construct was greater than its inter-construct correlations. Consequently, the constructs were deemed distinct. The HTMT values were significantly lower than the recommended cut-off value of 0.85, which further supports the validity of the discriminant function. This validation procedure is highly aggressive and guarantees the reliability and validity of the measurement model, thereby enabling it to be suitable for further analysis (Table 4).

**Table 4:** HTMT table (source: authors own creation).

Construct	ASU	CDE	CSA	EEN	LHU	PSA	REF	REM
ASU								
CDE	0.238							
CSA	0.356	0.744						
EEN	0.612	0.699	0.787					
LHU	0.665	0.653	0.695	0.758				
PSA	0.778	0.611	0.619	0.81	0.793			
REF	0.299	0.151	0.178	0.222	0.122	0.155		
REM	0.533	0.548	0.585	0.784	0.794	0.709	0.152	
SEP	0.371	0.884	0.637	0.736	0.727	0.606	0.289	0.541

### 5.3 Descriptive results

The R^2^ value is a measure of the extent to which exogenous constructs explain variance in endogenous constructs. Leadership behaviors and psychological needs fulfillment accounted for 65.9% of the variance in employee engagement, as indicated by an *R*^2^ value of 0.67 (Hair et al., 2021). Similarly, the *R*^2^ value for Service Performance was 0.57, indicating that 57% of the variance in performance was accounted for by Employee Engagement. These values suggest that the PLS-SEM model has very high explanatory power (Magno et al., 2022). The blindfolding technique was used to assess the predictive relevance of the model using the Q^2^ criterion. The model is perceived as predictively relevant, as the calculated Q^2^ value of the exogenous constructs exceeds zero (Hair et al., 2022). The model’s predictive relevance was demonstrated by the values of Q^2^ for Employee Engagement and Service Performance, respectively, which were 0.45 and 0.42, respectively (Chin et al., 2020).

Additionally, the practical implications of each predictor on endogenous constructs were assessed by calculating f^2^ effect sizes. Cohen (2013) believed that f ^2^ values of 0.02, 0.15, and 0.35 are indicative of minor, medium, and large effects, respectively (Magno et al., 2022). Employee Engagement in Service Performance exhibited the greatest effect size (f^2^ = 0.66) and substantial size. The effect sizes of other constructs were small to medium, indicating that they made a variety of contributions to the model. Recent advances have suggested using the standardized root mean square residual (SRMR) to assess model fit, although PLS-SEM normally does not use global goodness-of-fit metrics that are common with covariance-based SEM (Río-Ram). According to Magno et al. (2022), the SRMR value for this model should be at least 0.08; however, this is only 0.056. The results are supported by the fact that the model adequately describes and fits the data well, indicating an excellent match.

## 6 Discussion

The results show that H1, Leadership Humility (LHU) significantly influence Employee Engagement (EEN) and is supported at a significance level of (*β* = 0.224, *p* < 0.001). These results are consistent with the majority of empirical studies (Siachou et al., 2024; Wang et al., 2024) that emphasize humility as a fundamental leadership quality that is essential for establishing a positive work environment in which employees feel valued and capable of engaging in their work. Leadership humility probably allows for an environment of open collaboration through which employees can become better engaged and committed to their work.

The strong positive effect of REM on EEN (*β* = 0.186, *p* < 0.001) supports H2. This corresponds with previous studies (De La Cruz Del Río-Rama et al., 2020; Arathy and Biju, 2021) that have highlighted that empowerment increases employees’ motivation and inclination to participate in organizational activities. Relational empowerment creates feelings of ownership and responsibility and, hence, high engagement among employees.

In the model H3, Psychological Safety (PSA) was equally highly significant in impacting Employee Engagement (EEN) (*β* = 0.138, *p* = 0.003). This corresponds with previous studies (Quansah et al., 2023; Vakira et al., 2022) that the psychological safety of allowing individuals to freely open up without feeling any backlash enables them to become more engaged. Hence, a work environment must be developed in which employees feel sufficiently secure to take risks and express their ideas. These findings further support the role of SDT constructs in enhancing employee engagement.

ASU significantly influenced EEN (*β* = 0.135, *p* = 0.001), as proposed in H4. This result is consistent with prior research (Mokgata et al., 2022; Gu et al., 2021) that has indicated personal autonomy as a significant factor in the development of intrinsic motivation and a factor in engagement.

Finally, CDE had the most substantial effect on EEN (*β* = 0.345, *p* < 0.001) and, as a result, supported Hypothesis 5. This result is in line with studies (Monica and Krishnaveni, 2018; Wallo et al., 2020) that claim that chances to develop one’s abilities bring about higher engagement. These employees feel engaged and see possibilities for growth and development.

Finally, relatedness fulfillment (REF) significantly influenced employee engagement (EEN) (*β* = 0.126, *p* < 0.001), thus supporting Hypothesis 6. The results are in line with studies (Au et al., 2002) that generally support the hypothesis of SDT regarding the fulfillment of a need for relatedness or connectedness to others concerning enhancing engagement. When employees feel that they belong and are connected to the organisation and their coworkers, they are much more predisposed to become engaged in their work.

The results confirm that Employee Engagement (EEN) is positively related to Service Performance (SEP) (*β* = 0.757, *p* < 0.001), supporting Hypothesis 7. This validates the literature (Badru et al., 2022; Rasool et al., 2024) on the centrality of the link between engaged employees and higher service performance outcomes. Engaged employees tend to provide quality services, thereby improving an organisation’s overall performance.

Next, employee engagement (EEN) positively and significantly influenced customer satisfaction (CSA) (*β* = 0.406, *p* < 0.001), thus confirming Hypothesis 8. This result is also similar to previous research (Darvishmotevali et al., 2024; Rini and Kusumawardhani, 2023) that found that engaged employees have more positive experiences and customer satisfaction. Engaged employees go the extra mile and are more likely to provide superior services to customers, thereby affecting customer satisfaction. Such discretionary effort not only creates more positive service encounters but also strengthens customer trust and loyalty. Importantly, this highlights engagement as an internal driver that initiates a chain reaction leading toward better customer outcomes. Lastly, Service Performance (SEP) also plays a critical role in determining Customer Satisfaction (CSA) at *β* = 0.477, *p* < 0.001, and therefore Hypothesis 9 is also supported. This relationship tends to highlight that high service performance tends to ensure customer satisfaction, which is quite widely found in recent literature by Uppal et al. (2024), Dahri et al. (2024). From a mediating perspective, it can be said that employee engagement not only had a direct influence on satisfaction, but they also had an indirect influence on satisfaction through their influence on the service performance (*b* = 0.757, *p* < 0.001). In other words, engaged employees are better employees, who, in turn, are perceived as providing better quality of service by their customers - leading to satisfaction. This mediating pathway (EEN - SEP - CSA) supports the suggestion that engagement needs to be converted to measurable performance results for customers to be in a position to perceive value. If engagement provides the motivational foundation, it is the service performance that translated motivation into observable behaviors that can be assessed by the customer.

## 7 Theoretical contribution

This study contributes to the theoretical landscape by merging Self-Determination Theory (SDT) (Deci et al., 2017) with Leader-Member Exchange (LMX) (Graen and Uhl-Bien, 1995) in explaining how leadership behavior and fulfillment of psychological needs interact within the process that leads employees to be engaged in the Chinese service industry. The study further contributes through the study of multiple drivers of engagement LMX (leadership humility, relational empowerment, and psychological safety) and SDT (autonomy support, competence development, and relatedness fulfillment). Importantly, it shows that employee engagement is a major factor in which humble leadership plays an important role – one of these lesser-investigated traits in the literature on collectivist cultures, in which leadership humility enhances the engagement of employees. This insight adds to the current leadership theories of a value for humility in leadership, particularly within service and cultural-contextual areas, such as China (Khattak et al., 2023).

## 8 Managerial implications

The implications of this study are several for both managers and leaders in the Chinese service industry in the context of raising employee engagement and subsequent organizational performance. First, the positive effect of leadership humility, relational empowerment, and psychological safety on employee engagement indicates that leadership styles need to be shifted. Organisations need to develop humble, motivating, and empowering leaders who open channels of communication and show an interest in their staff. Respect and trust will naturally develop when leaders are being humble in understanding their limitation because they value the ability of others. Managers should put more emphasis on relational empowerment, which means that the employees should be empowered through participation in decision-making and provided with opportunities to experience some sense of control over their work. Just as important is the environment in which they can work being characterized by psychological safety, in which an employee feels that he or she can open up his or her ideas and concerns without the fear that his or her openness will backlash. Measures include leadership development programs that foster humility, practices of empowerment training, and measures at the team level supporting psychological safety through open dialogue and constructive feedback.

If autonomy support indicates engagement of employees, the development of competence, and fulfillment in relation to the other two principles, then quality work environments should be in a position where an organization develops conditions that will allow for autonomy through flexible working hours. Work should also be decentralized to allow for employee decisions and management in running personal projects. Professional development can lead to feelings of competence and efficacy in one’s job. Finally, an organizational sense of membership is needed. Management can achieve this by promoting team spirit with group activities, socials and reward programs that extol the virtues of individual and group achievements. This study shows that an emphasis on these factors contributes to increased employee engagement with enhanced service delivery and satisfaction to the customers. Our holistic approach toward the development and caring of employees enhances our productivity and enables us to compete effectively in the services marketplace.

## 9 Future study and limitations

This study contributes to the understanding of the influences of leadership behaviors and psychological needs fulfillment on employee engagement and performance outcomes in the Chinese service industry. It is worth stating that the present research has some limitations. While purposive sampling was conducted to target the relevant respondents, the generalization of findings across different populations may be somewhat elusive. Additionally, the cross-sectional nature of the study restricts the ability to infer causality between the variables. In addition to the service sector in China, cultural values, institutional systems, and industry may have the direct influence of humble leadership and psychological need-satisfaction on employee outcomes. This also reduces the generalizability of current results. It is important to know more about transnational dynamics in order to understand the degree to which these dynamics exist beyond market particularities in different industries and national contexts. Moreover, qualitative methodologies can help to realize nuance which would not be evident without research relying solely on quantitative methods. Future research may consider employing random sampling techniques and longitudinal designs to increase the robustness of the results, thereby providing in-depth insights into the long-term effectiveness of leadership behaviors on employee engagement and organizational outcomes.

Future research could expand the scope of this study by exploring different industries and cultural contexts to assess the generalisability of the findings. The current research could be further expanded by conducting comparative studies across different organizational contexts to explore how leadership styles and psychological needs fulfillment differ or influence employee engagement in other industries. Further, the addition of mediators such as organizational culture, job satisfaction, or employee well-being may explain more of the variance that exists between technology-enabled leadership in virtual teams on one side and engagement and performance on the other. Such future research would contribute to greater knowledge and understanding of leadership and employee engagement in various contexts.

## Data Availability

The original contributions presented in the study are included in the article/supplementary material, further inquiries can be directed to the corresponding author.
